# Bioequivalence study of low dose drospirenone/ethinyl estradiol 3 mg/0.03 mg film tablets under fasting conditions in Turkish healthy female subjects

**DOI:** 10.1002/prp2.1253

**Published:** 2024-07-24

**Authors:** Ahmet Inal, Zafer Sezer, Berna Uluözlü, Melih Oflas, Martin Reinsch, Wolfgang Martin, Mümtaz M. Mazicioglu, Selma Alime Koru

**Affiliations:** ^1^ Erciyes University Faculty of Medicine, Department of Pharmacology and Erciyes University Good Clinical Practice and Research Center Kayseri Turkey; ^2^ Biofarma İlaç Sanayi ve Ticaret A.Ş İstanbul Turkey; ^3^ Analytisches Zentrum Biopharm GmbH Berlin Germany; ^4^ Pharmakin Consulting Services UG Neu‐Ulm Germany; ^5^ Erciyes University Faculty of Medicine, Department of Family Medicine and Erciyes University Good Clinical Practice and Research Center Kayseri Turkey; ^6^ Ideal Contract Research Organisation Ankara Turkey

**Keywords:** bioavailability, Bioequivalance study, low dose drospirenone, low dose ethynil estradiol, pharmacokinetic

## Abstract

This bioequivalence research aims to evaluate the relative bioavailability and pharmacokinetic characteristics of ethinyl estradiol and drospirenone in the test preparation in comparison to the reference preparation during fasting conditions. A liquid chromatography method with tandem mass spectrometry was used to determine the concentrations of drospirenone and ethinyl estradiol in plasma. The pharmacokinetic parameters that were analyzed were the maximum plasma concentration (*C*_max_), time to achieve *C*_max_ (*t*_max_), elimination half life, and area under the concentration time curve of plasma (AUC_0‐*t*_, AUC_0‐∞_ for ethinyl estradiol, and AUC_0‐72h_ for drospirenone). Both the AUC and *C*_max_ parameters were determined to be between 80.00% and 125.00% (90% confidence intervals), which is the acceptable range. Based on the study findings, it was concluded that the test formulation, which includes 3 mg of drospirenone and 0.03 mg of ethinyl estradiol, demonstrated bioequivalence when compared to the reference formulation.

## Graphical abstract

Mean plasma concentrations following administration of test and reference drugs.

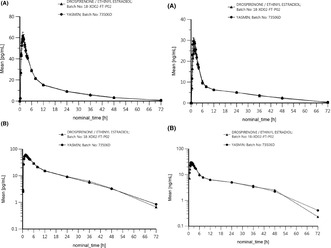

**Abbreviations**
AUC: area under the concentration time curve of plasma
*C*_max_
: maximum plasma concentration
CBG: corticosteroid
CI: confidence intervals
CRF: case report form
DRSP: drospirenone
ECG: electrocardiogram
EE: ethinyl estradiol
EMA: European Medicines Agency
FDA: U.S. Food and Drug Administration
GMP: good manufacturing practices
HIV: human immunodeficiency virus
HPLC: High‐performance Liquid Chromatography
LLOQ: lower limit of quantification
MS/MS: Mass Spectrometry
OTC: over‐the‐counter
R: reference drug
SHBG: sex hormone binding globulin
T: test drug
*t*_1/2_
: half life
TITCK: Turkiye Ilac ve Tıbbi Cihaz Kurumu
*t*_max_
: time to achieve *C*_max_

## 1 INTRODUCTION

The contraceptive efficacy of combined oral contraceptives results from the interplay of various factors, with primary emphasis on the ovulation inhibition and alterations in cervical secretion.^1^ Ethinyl estradiol (EE), a semi‐synthetic alkylated form of estradiol with a 17‐alpha‐ethinyl substitution, demonstrates high estrogenic potency when taken orally, often used as the estrogen component in oral contraceptives. Drospirenone (DRSP), a synthetic progestin analogous to spironolactone, is present in various oral contraceptive formulations. DRSP stands out from other synthetic progestins due to its pharmacological profile aligning closely with natural progesterone. Preclinical research on DRSP's pharmacological profile indicates that it approaches natural progesterone more than other synthetic progestins. Because of this, it is non‐androgenic, has anti‐mineralocorticoid characteristics, and inhibits the renin angiotensin aldosterone system's estrogen‐stimulated activity.^2^

When administered orally, DRSP is rapidly absorbed, reaching peak plasma concentration within 1–2 h. It has a bioavailability of approximately 76% and a half‐life ranging from 25 to 33 h. Steady‐state is achieved after seven once‐daily administration of DRSP 3 mg combined with 30 μg of EE. DRSP undergoes extensive metabolism, with no active metabolites, and is primarily bound to plasma albumin. Excretion is predominantly via feces, with slightly higher amounts than in urine. Metabolites, numbering at least 20, include glucuronide and sulfate conjugates.^3^

Due to presystemic conjugation and first‐pass metabolism, the absolute bioavailability of EE is approximately 45%. The excretion of metabolites has a half‐life of around 1 day. Twenty hours is the elimination half‐life. EE is not excreted unchanged. When EE conjugates with glucuronide and sulfate, it is eliminated through the enterohepatic circulation and is eliminated in the urine and feces. Furthermore, it has been demonstrated that EE leads to elevated blood concentrations of corticosteroid binding globulin (CBG) and sex hormone binding globulin (SHBG). Additionally, EE exhibits strong but nonspecific binding to serum albumin, with an approximate binding rate of 98.5%. Variations in the DRSP dose within the range of 2–3 mg had no influence on the effects of EE on SHBG and CBG.^4^

Conjugation with glucuronide or sulfate is the main metabolic pathway for EE and its oxidative metabolites. The primary oxidative process, 2 hydroxylation, is carried out by CYP3A4. Before being eliminated in the urine and feces, the 2‐hydroxy metabolite undergoes additional transformations, including methylation and glucuronidation.^5^, ^6^

For the needs of registration the efficacy and safety of any product are to be proved. There are two possibilities to do this for a new generic product: either by proving therapeutic equivalence or by proving bioequivalence with a market standard on the basis of comparison of relative bioavailability. The first option requires much higher numbers of patients and a long period of administration of either the test or the reference preparation. A bioequivalence trial on the basis of bioavailability is therefore generally accepted as the better alternative.^7^

Our study aims to explore the pharmacokinetic characteristics of DRSP and EE in healthy female volunteers with single dose oral administration under fasting conditions, assessing the bioequivalence of test versus reference tablets.

## 2 METHODS

### 2.1 Study design

The study was planned as a 2‐period crossover single‐dose bioequivalency study that was monocentric, randomized, open‐label, and intended to be carried out in healthy female volunteers under fasting conditions. The primary aim of the study was to assess the bioequivalence of the film tablets manufactured by Biofarma Turkiye, which include 3 mg of DRSP and 0.03 mg of EE. This comparison was conducted against the reference product Yasmin® 3 mg/0.03 mg Film tabletten, produced by Bayer AG‐Germany and distributed by Jenapharm GmbH & Co. KG‐Germany. Between January 11, 2019, and February 7, 2019, a screening process was conducted for a group comprising 50 female subjects of Caucasian ethnicity. Thirty‐three healthy female volunteers were enrolled, and 30 completed the study. The volunteers were administered the test and reference products in two distinct treatment regimens (sequence TR or RT) with a minimum of 14‐day washout time. They were required to stay at the clinical center for one night throughout each regimen, starting from the day before dosing and ending 12 h after treatment. Blood samples were taken ambulatorily at 24, 36, 48, and 72 h after the medication was administered.

The clinical part was not blinded, given that the main target criteria were pharmacokinetic parameters derived from plasma concentrations. However, researchers involved in the analytical phase remained blinded regarding the treatments.

Before the first dose, within 9 days, subjects underwent a physical examination, laboratory blood investigations, a 12‐lead ECG, and drug screening in urine. The screening process involved an assessment of lifestyle, habits, birth date, ethnicity, gender, medical and surgical history, body temperature, heart rate and supine blood pressure. Pregnancy tests were conducted on the screening day and during hospitalization for both periods. Nursing mothers or those using hormonal contraception were excluded. The use of prescription medication within 2 weeks, as well as over‐the‐counter (OTC) medication within 1 week of the study's start date, was an exclusion criterion. Concomitant medication, including herbal therapies, was generally prohibited, except when deemed critical for the subject's well‐being.

Inclusion criteria comprised female subjects aged 18 to 40, providing informed consent, with a BMI within the normal range (18.5 to 30 kg/m^2^). Exclusion criteria for volunteers included a history of gastrointestinal, renal, and liver disease, clinically significant abnormalities in hematology, known hypersensitivity to EE, DRSP, or related medications, as well as positive test results for hepatitis C virus, hepatitis B surface antigen, and/or human immunodeficiency virus (HIV).

The study adhered to ethical guidelines, including the Declaration of Helsinki, Turkish Pharmaceutical and Medical Preparations Law No. 1262, ICH‐GCP, and the Turkish Good Clinical Practice Guideline. The Ethics Committee at Erciyes University (Biyoyararlanim ve Biyoesdegerlik Etik Kurulu) and the Turkiye Ilac ve Tıbbi Cihaz Kurumu (TITCK) of the Turkish Ministry of Health gave their approval. Each subject signed their written informed permission before any trial‐related operations could begin, and each participant received one copy of the informed consent form.

### 2.2 Study drugs

The test product, Drospirenone/Ethinyl estradiol 3 mg/0.03 mg film tablet (T), batch no: 18‐XD02‐FT‐P02, expiry date: 09/2020, was produced under Good Manufacturing Practices (GMP) conditions by Biofarma at their facilities in Sancaktepe, Istanbul, Turkiye. The innovative product, Yasmin® 3 mg/0.03 mg filmtabletten (film tablets) (R), which was acquired from the German market, served as the reference preparation.

### 2.3 Drug administration and blood sampling

The evening before drug administration, volunteers were confined to the Erciyes University Good Clinical Practice and Research Center, IKUM (Centre for GCP) in Kayseri, Turkiye. Volunteers were given an evening snack around 9:00 p.m. after being admitted to the clinic, which included approximately 600 kcal of calories. Following a minimum 10 h overnight fast, 240 mL of drinking water were used to orally administer either the test preparation or the reference preparation, each containing 3/0.03 mg of DRSP/EE.

Blood samples were collected at various time points: 0:00 (pre‐dose), as well as at 15, 30, 45 min, 1 h, 1 h 15 min, 1 h 30 min, 1 h 45 min, 2 h, 2 h 30 min, 3 h, 3 h 30 min, 4 h, 6 h, 8 h, 12 h, 24 h, 36 h, 48 h, and 72 h post‐dose for pharmacokinetic analysis. This resulted in a total of 40 blood samples for each volunteer across 2 periods, with each sample being 7 mL. The total blood loss per volunteer was approximately 280 mL, excluding the initial and final safety laboratory examinations. After the drug administration, there was lunch and dinner served 4 and 10 h later, respectively. During sample days, each subject's intake of food, water, and physical exercise was standardized. Fruit juices and products containing xanthine were forbidden while the patient was in the clinical center.

Blood samples (20 in each period) were taken by a short intravenous catheter, collecting 7 mL into K_2_‐EDTA tubes and cooled immediately at 2–8°C in a mini‐refrigerator. The blood samples were centrifuged within 20 min after sampling for 10 min at 3000 × g under refrigeration (4–7°C) to obtain plasma. Following centrifugation, the plasma that was separated from each sample was placed into two transparent, 3.5 mL polypropylene tubes for each sample (with a minimum of 1.5 mL per tube). These tubes were then stored at <−70°C and sent to Analytisches Zentrum Biopharm GmbH Berlin, Germany, on dry ice.

### 2.4 Bioanalytical method

Following the guidelines of the European Medicines Agency (EMA)^8^ and the U.S. Food and Drug Administration (FDA),^9^ a LC–MS/MS method was used in March 2019 for the validation of DRSP and EE. The derivatization of EE with dansyl chlorid was performed according literature.^10^ Nevertehless, a new gradient chromatographic method was developed by the analytical facility due to the simultanoues determination of EE and DRSP while EE was formerly determined using an isocratic assay in the literature.^10^ The validation aimed to demonstrate selectivity in plasma, intra‐assay and inter‐assay precision and accuracy, lower limit of quantification (LLOQ), dilution integrity, carryover, recovery, matrix effect, linearity of the standard curve (ranging from 2.00 to 300.00 pg/mL for EE and 2.50 to 375.00 ng/mL for DRSP), and stability data under various conditions (benchtop, freeze–thaw, autosampler, extract stability, and long‐term stability). The outcomes showed that it was possible to measure DRSP and EE in plasma samples with a high enough degree of specificity, sensitivity, accuracy, and precision.

DRSP and EE in plasma samples were determined using the validated technique, which combined High‐Performance Liquid Chromatography (HPLC) with Mass Spectrometry (MS/MS) detection. DRSP, EE, and their stable isotopic labeled internal standards drospirenone‐d_4_ and ethinyl estradiol‐d_4_ were separated using a gradient condition with acetonitrile and formic acid in water, utilizing a Waters XSelect® HSS T3 HPLC column. Analyst (1.6.2) chromatography software was used to get chromatographic data. The sequence files and technique were set up before the first sample was injected. Analyte/internal standard peak area ratios were plotted against corresponding standard concentrations to create standard curves for each test day. By comparing peak area ratios in the sample to the weighted (1/x) regression equation derived from standard data, sample concentrations were calculated analytically. The validated program dbLabCal was used to carry out the calibration function and the sample concentration computation.

### 2.5 Pharmacokinetic evaluation and statistics

Individual pharmacokinetic parameters for EE were determined, including AUC_(0‐*t*)_ and AUC_(0‐∞)_, *C*_max_, *t*_max_, *λ*_z_, *t*_½(λz)_, and AUC_%‐extrapolation_. Similarly, pharmacokinetic parameters for DRSP, such as AUC_(0‐72h)_ and AUC_(0‐*t*)_, *C*_max_, *t*_max_, *λ*_z_, *t*_½(λz)_, and AUC_%‐extrapolation_, were also determined. The primary outcomes were defined as the intraindividual bioavailability ratios in the measurements of AUC_(0‐*t*)_ and *C*_max_ for EE, as well as AUC_(0‐72h)_ and *C*_max_ for DRSP, following administration of a single test dosage compared to the reference formulation. Bioequivalence was considered achieved if the acceptance criteria of 90% confidence intervals (CI) (80.00%–125.00%) for the test (T) versus reference (R) comparisons were met for all primary endpoints. Secondary target variables included additional pharmacokinetic measures after single‐dose administration (AUC_(0‐∞)_, *t*_max_, *λ*_z_, *t*_½(λz))_. AUC_%‐extrapolation_ was provided for the examination of the extrapolation process. The pharmacokinetic evaluation was conducted using the Phoenix WinNonlin V8.1.0.3530 program for noncompartmental assessment of data. The slopes of log (concentration)/time curves (*λ*_z_) were determined based on the last four available data points above LLOQ. Non‐parametric evaluations using two one‐sided Wilcoxon tests were applied for *t*_max_.

### 2.6 Safety assessment

Any adverse event (AE) that was reported voluntarily by the study participants, revealed during standard inquiries upon admission to the clinical unit prior to the first and second study periods, or recorded at particular study time points (0, 1, 6, 12, 24, 36, 48 and 72 h) during both periods was carefully recorded on an AE information sheet that was included in the subject's Case Report Form (CRF). In addition, throughout the first and final examinations, blood pressure (both systolic and diastolic) and heart rate were recorded. Every participant had a single electrocardiogram (ECG) taken during the admission and final examinations. The CRFs for each participant contain the results of their individual measures of heart rate and blood pressure (both diastolic and systolic).

### 2.7 Nomenclature of targets and ligands

Key protein targets and ligands in this article are hyperlinked to corresponding entries in http://www.guidetopharmacology.org, the common portal for data from the IUPHAR/BPS Guide to PHARMACOLOGY,^11^ and are permanently archived in the Concise Guide to PHARMACOLOGY 2019/20.^12^

## 3 RESULTS

### 3.1 Pharmacokinetic results

A physical examination and clinical laboratory testing were used to assess 50 female Caucasian volunteers in total. Thirty‐three of the healthy female individuals were included to the research. Thirty volunteers with a BMI ranging from 18.6 kg/m^2^ to 29.7 kg/m^2^, ages 18 to 38, eventually finished the trial in accordance with the study protocol. As such, plasma samples from 30 patients that successfully finished each therapy were available for the determination of the amounts of DRSP and EE. The mean (± s.d.) demographic data for the 30 completed cases were as follows: 27.4 (± 6.2) years in age, 64.1 (± 11.2) kg in weight, 161.3 (± 5.1) cm in height, and a BMI of 24.6 (± 3.9) kg/m^2^. Table 1 is a summary of the demographic data.

**TABLE 1:** A summary of the demographic data.

Parameter	Age (a)	Weight (kg)	Height (cm)	BMI (kg/m^2^)
Mean value	27.4	64.1	161.3	24.6
Standard deviation	6.2	11.2	5.1	3.9
CV (%)	22.6	17.5	3.2	15.9
Minimum	18	47.0	148	18.6
Maximum	38	89.0	173	29.7
Number	30	30	30	30

There were noticeable similarities between the test and reference drugs when pharmacokinetic data were compared. As shown in Table 2, there were no significant differences in the EE characteristics, such as AUC_(0‐*t*)_, AUC_(0‐∞)_, and *C*_max_. Similarly, for DRSP, both AUC_(0‐72)_ and *C*_max_ exhibited a high degree of similarity between the test and reference products, as outlined in Table 3.

**TABLE 2:** Ethynil estradiol pharmacokinetic characteristics (arithmetic means ± standard deviation); *n* = 30 volunteers.

Drugs	AUC_(0‐*t*)_ [μg × h/mL]	AUC_(0‐∞)_ [μg × h/mL]	*C*_max_ [ng/mL]	*t*_max_ [h]	*λ*_z_ [1/h]	*t*½ (*λ*z) [h]	%AUC_extrapolation_ [%]
T:Drospirenone/ethinyl estradiol 3 mg/0.03 mg film tablet batch number: 18‐XD02‐FT‐P02	685.47 ± 231.23	785.81 ± 275.23	65.28 ± 18.03	1.57 ± 0.59	0.0433 ± 0.0157	17.95 ± 5.91	12.26 ± 5.69
R: Yasmin® 3 mg/0.03 mg filmtabletten (film tablets) batch number: 73506D	679.26 ± 226.04	764.69 ± 294.05	63.41 ± 14.09	1.62 ± 0.45	0.0448 ± 0.0138	17.15 ± 6.36	10.41 ± 3.85

**TABLE 3:** Drospirenone pharmacokinetic characteristics (arithmetic means ± standard deviation); *n* = 30 volunteers.

Drugs	AUC_(0‐*t*)_ [μg × h/mL]	AUC_(0‐72)_ [μg × h/mL]	*C*_max_ [ng/mL]	*t*_max_ [h]	*λ*_z_ [1/h]	*t*½ (*λ*z) [h]	%AUC_extrapolation_ [%]
T: Drospirenone/ethinyl estradiol 3 mg/0.03 mg film tablet batch number: 18‐XD02‐FT‐P02	306.57 ± 108.92	329.23 ± 111.59	29.17 ± 6.80	1.92 ± 0.81	0.0325 ± 0.0514	33.46 ± 13.97	33.36 ± 11.11
R: Yasmin® 3 mg/0.03 mg filmtabletten (film tablets) batch number: 73506D	316.13 ± 104.43	333.78 ± 109.11	34.10 ± 7.02	1.47 ± 0.50	0.0265 ± 0.0198	34.18 ± 16.71	31.76 ± 10.67

**FIGURE 1:**
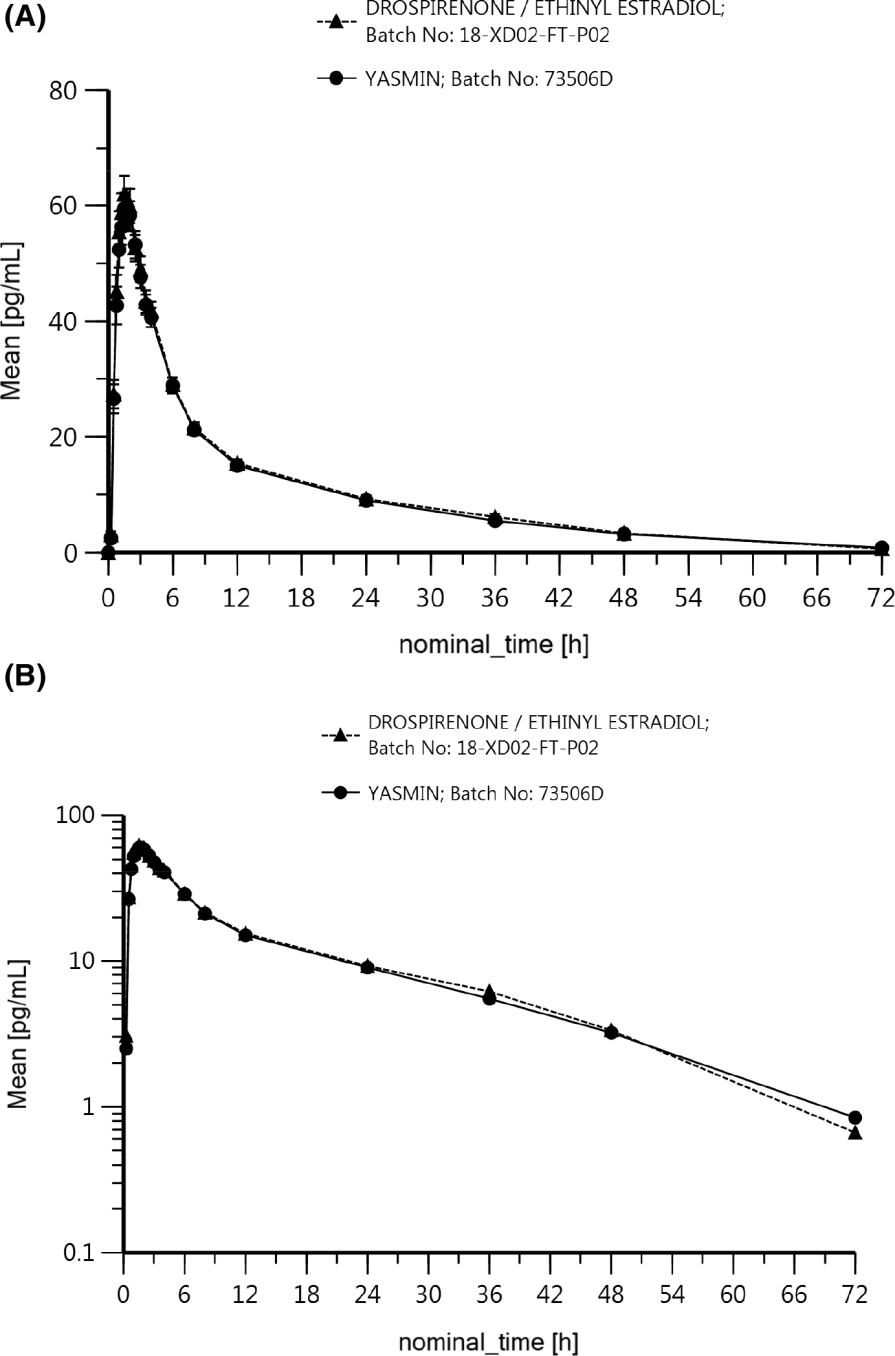
(A) Mean (± standard deviation) Ethynil estradiol plasma concentrations following administration of various (test and reference) products; linear plot; *n* = 30. (B) Mean Ethynil estradiol plasma concentrations following administration of different (Drospirenone/Ethinyl estradiol and Yasmin) drugs; loglinear plot; *n* = 30.

**FIGURE 2:**
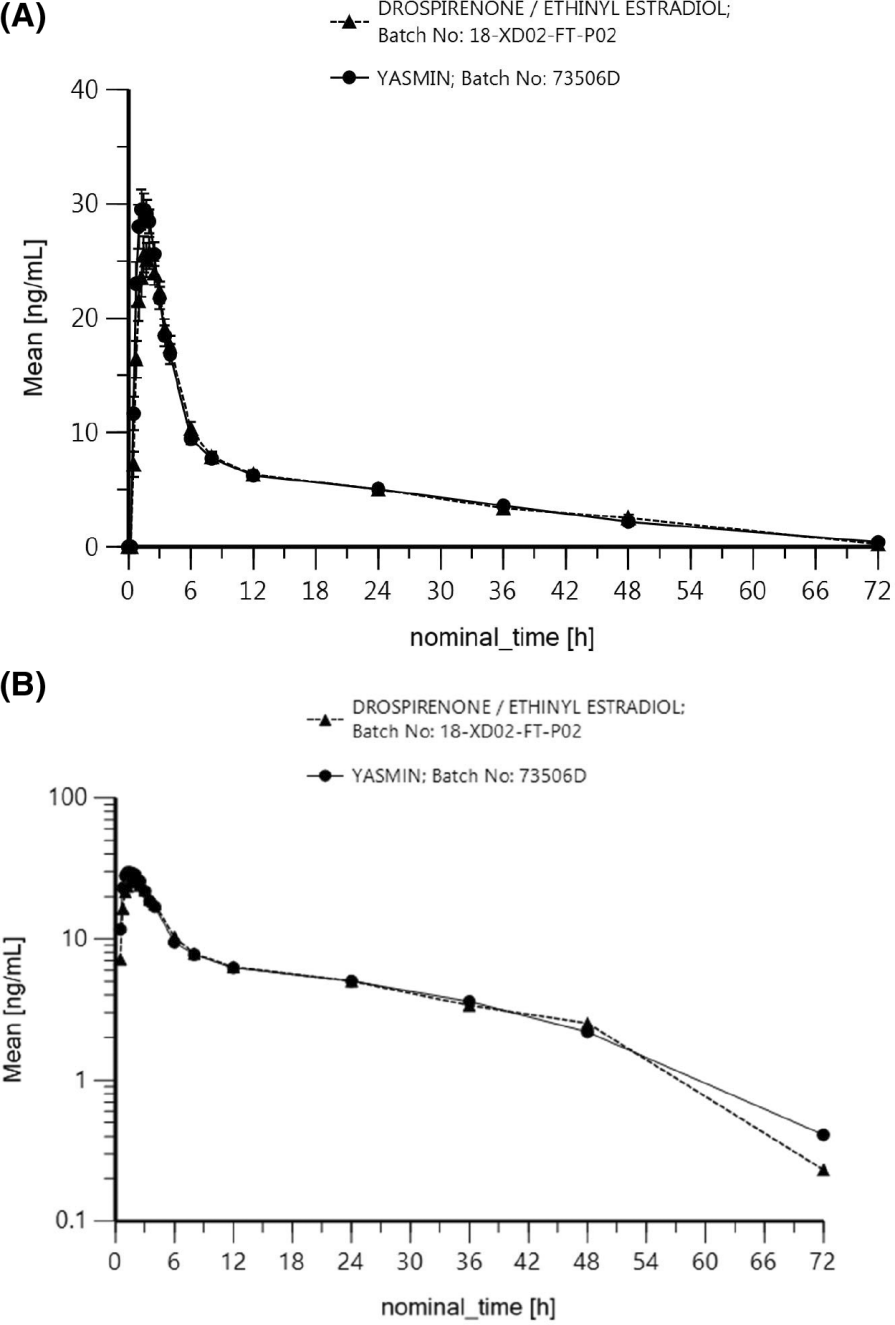
(A) Mean Drospirenone plasma concentrations (± sem) following administartion of different (Drospirenone/Ethinyl estradiol and Yasmin) drugs; linear plot; *n* = 30. (B) Mean Drospirenone plasma concentrations following administration of different (Drospirenone/Ethinyl estradiol and Yasmin) drugs; loglinear plot; *n* = 30.

For a more comprehensive understanding, Figures 1A,B and 2A,B present mean plasma concentration‐time profiles for EE and DRSP across different time points and treatments. These graphical representations visually confirm the comparable pharmacokinetic profiles observed between the test and reference drugs. In summary, the data from both tables and figures collectively support the assertion of bioequivalence between the test and reference drugs.

The 90% confidence intervals for the ratios of geometric means for the test and reference formulations were determined using ANOVA and two one‐sided t‐tests for the primary pharmacokinetic target variables, which included AUC_(0‐*t*)_ and *C*_max_ for EE, as well as AUC_(0‐72h)_ and *C*_max_ for DRSP. These calculations were based on the assumption of a lognormal distribution of the individual data, and they were performed to assess the bioequivalence between the test and reference drugs.

Secondary pharmacokinetic variables, such as AUC_(0‐∞)_, t_max_ for EE, and *t*_max_ along with the exploratory parameter AUC_(0‐*t*)_ for DRSP, were considered as supportive data. AUC_(0‐∞)_ was evaluated using a multiplicative model similar to AUC_(0‐*t*)_, while *t*_max_ underwent nonparametric analysis employing 90% confidence intervals derived from two one‐sided Wilcoxon test.

The observed 90% confidence intervals, along with the corresponding point estimators (ratios of test/reference geometric means), are presented in Tables 4 and 5. These analyses contribute to the comprehensive evaluation of bioequivalence between the DRSP/EE and Yasmin, providing insights into the primary and secondary pharmacokinetic parameters.

**TABLE 4:** Statistical results of ethinyl estradiol (test vs reference); *n* = 30 subjects.

Pharmacokinetic variables: (ANOVA; two one‐sided *t*‐tests)	90% confidence intervals	point estimators (geometric means; ratios test/reference)	Intra subject variability
AUC_(0‐*t*)_	95.71%–106.49%	100.96%	12.19%
*C*_max_	96.73%–106.85%	101.67%	11.36%
AUC_(0‐∞)_	98.40%–108.27%	103.22%	10.92%

**TABLE 5:** Statistical results of Drospirenone (test vs reference); *n* = 30 subjects.

Pharmacokinetic variables: (ANOVA; two one‐sided *t*‐tests)	90% confidence intervals	Point estimators (geometric means; ratios test/reference)	Intra subject variability
AUC_(0‐72h)_ ^a^	92.71%–102.15%	97.32%	10.45%
*C*_max_	80.18%–89.99%	84.95%	13.20%
AUC_(0‐*t*)_	91.30%–99.76%	95.44%	10.12%

^a^ *n* = 27.

### 3.2 Safety results

Throughout the study, there were 26 recorded adverse events in all, affecting 14 out of the 33 enrolled volunteers. The adverse events comprised 6 instances of headache, 5 cases of vomiting, 5 incidents of menstrual bleeding, 3 reports of nausea, 2 occurrences of increased menstrual bleeding, 2 instances of delayed menstrual cycles, and 1 each of palpitation, dizziness, and disappetite.

Of these, 11 adverse events were classified as mild in intensity (7 of them are test, 4 of them are reference), while 15 were of moderate intensity (6 of them are test, 9 of them are reference). A single 500 mg dosage of paracetamol was administered to each of the four adverse events (1 headache in the test preparation during period I); 3 headaches in the reference preparation (1 during period I and 2 during period II) in accordance with the study protocol. Notably, all observed adverse events resolved without any lasting effects. The assessment of the drug relationship for all treatment‐emergent adverse events was consistently categorized as “possible” (26 out of 26).

Specifically, 13 treatment‐emergent adverse events occurred after administration of the test preparation, encompassing 1 mild and 2 moderate instances of menstrual bleeding, 1 mild and 2 moderate cases of headache, 1 moderate instance of increased menstrual bleeding, 1 mild and 1 moderate episode of delayed menstrual cycle, and 1 each of mild nausea, mild vomiting, mild palpitation, and mild dizziness.

Similarly, 13 treatment‐emergent adverse events were noted after the reference preparation, including 1 mild and 3 moderate cases of vomiting, 3 moderate instances of headache, 1 mild and 1 moderate case of nausea, 1 mild and 1 moderate episode of menstrual bleeding, 1 moderate incident of increased menstrual bleeding, and 1 mild case of disappetite.

Crucially, no severe or serious adverse events were recorded following treatment with either the test or reference preparation. All reported adverse events resolved without any lasting effects.

## 4 DISCUSSION

A pharmaceutical product's therapeutic efficacy is largely dependent on its bioavailability, which is determined by the rate and amount of drug absorption.^13^ According to guideline on the “Investigation of Bioequivalence,” two pharmaceuticals are considered bioequivalent when, following the administration of the same molar dose, their bioavailabilities are so comparable that their effects on safety and effectiveness are nearly equal.^7^ When the 90% confidence intervals of AUC and *C*_max_ T/R ratio are between 80.00% and 125.00%, the condition is satisfied. The findings of our study indicate that the extent (AUC_(0‐*t*)_ and AUC_(0‐72h)_) and rate (*C*_max_) of bioavailability for the test formulation (Drospirenone/ethinyl estradiol 3 mg/0.03 mg Film Tablet) and reference formulation (Yasmin® 3 mg/0.03 mg filmtabletten) are comparable.

Both the ln‐transformed AUC_(0‐*t*)_ and ln‐transformed *C*_max_ for EE and the ln‐transformed AUC_(0‐72h)_ and *C*_max_ for DRSP have 90% confidence intervals that satisfy the bioequivalence requirements of 80.00%–125.00%. Additionally, the 90% confidence intervals of ln‐transformed data for the secondary parameter AUC_(0‐∞)_ of EE and the exploratory parameter AUC_(0‐*t*)_ of DRSP also fall within the range of acceptability for bioequivalence. The tolerability and safety of the therapies did not vary in any way that was clinically significant, according to the research.

Bioequivalence between Drospirenone/ethinyl estradiol 3 mg/0.03 mg film tablet and Yasmin® 3 mg/0.03 mg filmtabletten has been demonstrated for AUC_(0‐*t*)_ and *C*_max_ of ethinyl estradiol (*n* = 30 subjects) and AUC_(0‐72h)_ and *C*_max_ of DRSP (*n* = 27 subjects for AUC_(0‐72h)_, *n* = 30 subjects for *C*_max_) in healthy female caucasian volunteers following single dose drug administration under fasting conditions.

The *C*_max_ and AUC_(0‐tlast)_ of EE in this investigation have observed geometric mean ratios (test to reference) of 100.96% and 101.67%, respectively. The observed ratios for *C*_max_, AUC_(0‐72h)_, and AUC_(0‐*t*)_ for DRSP were 84.95%, 97.32%, and 95.44%, respectively.

Given that this combination of EE and DRSP is intended for female contraception, only female participants were included in the study, representing the target population. The Summary of Product Characteristics (SmPC) for the reference product does not specify whether the product should be taken with or without food. According to guideline, the study was appropriately planned under fasting conditions, eliminating the need for repetition under fed conditions.^7^

Ethinyl estradiol/drospirenone may potentially cause adverse reactions in some individuals. Some of the common adverse reactions associated with ethinyl estradiol/drospirenone are blood clots, including deep vein thrombosis, pulmonary embolism, stroke, heart attack, liver problems, hypertension.^14^ In our study, low dose administration of the drug in healthy volunteers reduced these risks.

However, it's acknowledged that the study population size might limit the safety information obtained. Recommendations for further phase 4 studies and the establishment of a comprehensive pharmacovigilance system are advised to enhance the safety profile of the product.

## 5 CONCLUSION

This study, conducted with healthy fasting Caucasian adult female subjects, provides compelling evidence that the combination of EE and DRSP in the test formulation meets the stringent regulatory requirements of both Turkish and European authorities for establishing bioequivalence. The strong pharmacokinetic, statistical, and safety results indicate that the test formulation is equivalent to the reference product.

Encouraging the development and availability of generic products, exemplified by this study, aligns with global initiatives aimed at fostering cost‐effective alternatives. Such endeavors contribute to the broader goal of enhancing accessibility within healthcare systems, ensuring that effective treatments are available to a wider population.

## AUTHOR CONTRIBUTIONS

All authors contributed to the concept and design of the study. Berna Ulu Özlü and Melih Oflas took decisions in the name of the sponsor. Selma Koru prepared study documentation and carried out the necessary application and submissions for the study. Ahmet Inal carried out the clinical part as the principal investigator, Zafer Sezer and Mümtaz Mazıcıoğlu as co‐investigator. Martin Reinsch, performed Bioanalysis in plasma. Wolfgang Martin performed the Pharmacokinetic analysis, statistics and reporting parts. Ahmet Inal and Selma Koru contributed to writing the manuscript. All authors approved the final version of the manuscript.

## CONFLICT OF INTEREST STATEMENT

There are no conflicts of interest reported by any of the authors.

## Data Availability

The data are not publicly available due to compromise the privacy of research participants and due to the fact that trade secret. The data that are publicly available are shared in the article.
